# MotP Subunit is Critical for Ion Selectivity and Evolution of a K^+^-Coupled Flagellar Motor

**DOI:** 10.3390/biom10050691

**Published:** 2020-04-29

**Authors:** Shun Naganawa, Masahiro Ito

**Affiliations:** 1 Graduate School of Life Sciences, Toyo University, Oura-gun, Gunma 374-0193, Japan; success630408@gmail.com; 2 Bio-Nano Electronics Research Centre, Toyo University, 2100 Kujirai, Kawagoe Saitama 350-8585, Japan

**Keywords:** *alkaliphiles*, Mot complex, potassium ion, flagellar motor, evolution, *Bacillus*

## Abstract

The bacterial flagellar motor is a sophisticated nanomachine embedded in the cell envelope. The flagellar motor is driven by an electrochemical gradient of cations such as H^+^, Na^+^, and K^+^ through ion channels in stator complexes embedded in the cell membrane. The flagellum is believed to rotate as a result of electrostatic interaction forces between the stator and the rotor. In bacteria of the genus *Bacillus* and related species, the single transmembrane segment of MotB-type subunit protein (MotB and MotS) is critical for the selection of the H^+^ and Na^+^ coupling ions. Here, we constructed and characterized several hybrid stators combined with single Na^+^-coupled and dual Na^+^- and K^+^-coupled stator subunits, and we report that the MotP subunit is critical for the selection of K^+^. This result suggested that the K^+^ selectivity of the MotP/MotS complexes evolved from the single Na^+^-coupled stator MotP/MotS complexes. This finding will promote the understanding of the evolution of flagellar motors and the molecular mechanisms of coupling ion selectivity.

## 1. Introduction

Many motile bacteria have a spiral flagellum as a locomotor and move in the environment by rotating one or more flagellar bundles. The bacterial flagellar motor is a high-performance nanomachine rotating at high speed [1,2,3]. The flagellum is rotated in the counterclockwise direction for smooth swimming and is rotated in the clockwise direction to change the direction [4,5]. Bacterial flagella consist of three parts: a base body corresponding to the motor part embedded in the membrane, flagellar filament equivalent to a propeller extended long outside of the cell body, and a hook connecting the base body and flagellar filament [6]. Bacterial flagella are composed of approximately 30 kinds of proteins, and they form a supercomplex [7]. The basal body of the flagellar motor consists of a rotor and a stator. The electrostatic interaction between the rotor and the stator is the driving force of flagellar rotation [4,8,9,10]. The stator complex is composed of two subunits (MotA-type and MotB-type) formed at a ratio of 4:2, functions as an ion channel and anchors to the cell wall through a putative peptidoglycan-binding (PGB) motif in the periplasmic domain of a MotB-type protein. Mot complexes are arranged in a ring of membrane-embedded complexes surrounding each flagellum [1,2]. A typical number of such complexes surrounding the basal motor appears to be at least 11 [11]. In general, *Escherichia coli* MotA/MotB complex is an H^+^ driven flagellar motor. In contrast, marine *Vibrio* species PomA/PomB complex and alkaliphilic *Bacillus* MotP/MotS complex are Na^+^ driven flagellar motors, respectively [12,13,14,15]. Each stator complex has homology to each other. *E. coli* and alkaliphilic *Bacillus pseudofirmus* have only one type of Mot complex in the motor [16,17,18]. On the other hand, *Bacillus subtilis* and *Shewanella oneidensis* can have two distinct Mot complexes in the motor [19,20]. However, in 2008, the alkaliphilic *Bacillus clausii* KSM-K16 was identified as the first bacterium to have a single stator rotor that uses both H^+^ and Na^+^ for ion coupling depending on the Ph [21]. Mutations that convert the bifunctional stator to each single stator type have been demonstrated, and the same approach was utilized to generate dual-ion use stators from the two single ion-use stators of *B. subtilis* [21]. Subsequent findings have shown that alkaliphilic *Bacillus alcalophilus* AV1934 uses Na^+^, K^+^, and Rb^+^ [22] and that *Paenibacillus* sp. TCA20 uses Ca^2+^ and Mg^2+^ as coupling ions for flagellar rotation [3,23].

Previously, the transmembrane segment of the MotB-type subunit was proposed to be critical for the coupling ion selectivity of the stator when H^+^ or Na^+^ were used as coupling ions [24,25]. At the N-terminal side of the single transmembrane region of the MotB-type subunit, there is an aspartic acid residue that functions as a universally conserved putative coupling ion binding site [26]. The amino acid residue located ten amino acids downstream from the aspartic acid residue is presumed to be critical for coupling ion selectivity [21]. The amino acid residue at position is highly conserved as a valine residue in the H^+^-coupled MotB subunit and a leucine residue in the Na^+^-coupled MotS and PomB subunits [21]. However, it has been reported that the *B. alcalophilus* Na^+^- and K^+^-coupled MotS subunit is conserved as a methionine residue (MotS_Met33) at the same position, and the MotS_Met33Leu substituted stator of *B. alcalophilus* lost its original potassium-coupling capacity. Therefore, the methionine residue is critical for K^+^ selectivity [22].

There have been no reports of bacteria that can utilize both Na^+^ and K^+^ as the coupling ions for a flagellar motor except *B. alcalophilus*. It is premature to draw conclusions about all the K^+^ selection mechanisms in one reported example. Details on the coupling of the K^+^ selective mechanism are still poor. Finding another example that can utilize K^+^ for flagellar rotation is important for elucidating the coupling ion selectivity mechanism. Therefore, we investigated novel bacteria that can use K^+^ as a coupling ion for a flagellar motor and identified the stator of the alkaliphilic bacterium *Bacillus trypoxylicola*, which was isolated from the intestines of Japanese beetle larvae [27]. This bacterium was the second example that could utilize both Na^+^ and K^+^ as a coupling ion for the flagellar motor.

In this study, we analyzed the differences in the ion selectivity mechanisms of the flagellar motor stator between the single Na^+^-coupled and dual Na^+-^ and K^+^-coupled stators.

## 2. Materials and Methods 

### 2.1. Bacterial Strains and Plasmids

The bacterial strains and plasmids used in this study are listed in Table 1 and Table 2, respectively. The primers used in this study are listed in Table S1.

### 2.2. Cloning of the motP/motS Genes with the Pre- and Post-Regions of B. trypoxylicola

**Figure 1:**
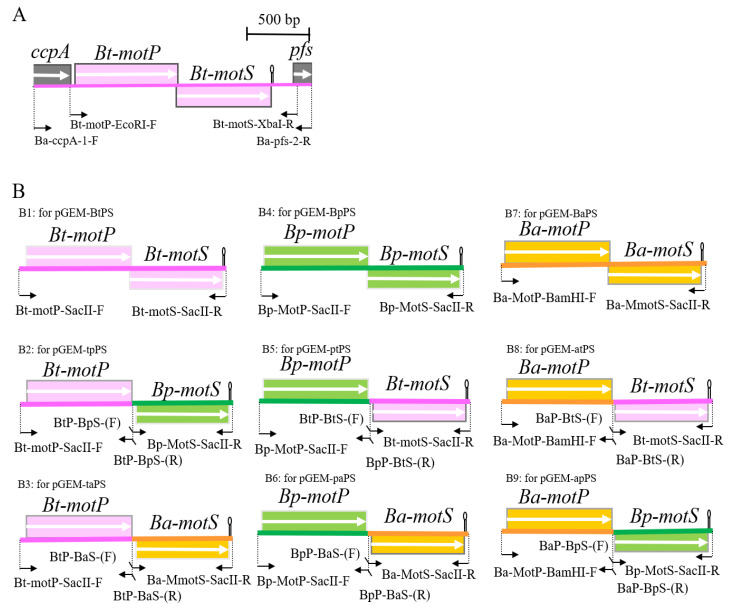
Schematic diagram of the primers used for hybrid stator construction and PCR. (**A**) Schematic diagram of the *motP/motS* locus of *B. trypoxylicola* chromosome and the primers used for PCR. (**B**) Schematic diagram of amplification of *Bt-motP/motS*, BP-*motP/motS*, *Ba-motP/motS*, and a series of hybrid *motPS* that indicate the primers used for cloning downstream of *P_xylA_* promoter of integration plasmid pAX01. The detailed protocol for construction of each PCR product is described in Materials and Methods. The mark after each *motS* gene indicates the position of the terminator.

Since *B. trypoxylicola* NBRC102646 has no whole-genome sequence information at the beginning of the study in 2014, the primers used for PCR were designed based on the gene sequence in the region before and after the closely related *B. alcalophilus* stator gene. The *B. trypoxylicola motPS* genes were amplified by PCR using *B. trypoxylicola* chromosomal DNA as the template with Ba-ccpA-1-F and Ba-acuC-2-R primers (Figure 1A and Figure S1). GoTaq Green Master Mix (Promega, Madison, Wisconsin, USA) was used for PCR, and the reaction was performed according to the manufacturer’s protocol. The amplified 2215 bp PCR product was purified using a QIAquick Gel Extraction Kit (QIAGEN, Hinden, Germany) and ligated to a pGEM-T Easy Vector (Promega) using T4 DNA Ligase (New England Biolabs, Ipswich, MA, USA). The ligation reaction and composition were performed according to the instructions. The reaction solution was added to competent *E. coli* DH5α MCR cells prepared by the rubidium chloride method [34], and transformation was performed by the heat-shock method. The transformed cells were spread on LB plates supplemented with ampicillin to a final concentration of 100 μg/mL and cultured at 37 °C overnight. The desired plasmid was extracted from the colonies on the plates by a QIAprep Spin Miniprep Kit (QIAGEN, Germany) according to the instructions, and the desired plasmid was obtained and named pGEM-T-BtPS. In the following molecular biology experiments, the same method as described above was used unless otherwise specified.

Next, the *B. trypoxylicola motPS* genes were amplified by PCR using pGEM-T-BtPS as a template with the Bt-motP-EcoRI-F and Bt-motS-XbaI-R primers (Figure 1A and Figure S1). Phusion High-Fidelity DNA Polymerase (New England Biolabs, Ipswich, MA, USA) was used for PCR. The PCR composition and reaction conditions were performed in accordance with the Phusion High-Fidelity DNA Polymerase protocol. The amplified 2,123 bp PCR product was purified, and the PCR product was ligated to *Sma*I-digested pGEM7zf (+). The ligation mixture was transformed into *E. coli* DH5α MCR, and the transformed cells were spread on LB plates supplemented with ampicillin to a final concentration of 100 μg/mL and cultured at 37 °C overnight. The desired plasmid was obtained and named pGEM-BtPS. The DNA sequence was deposited into DNA Data Bank of Japan (DDBJ), and the accession number is LC532380.1.

### 2.3. Cloning of the motP/motS Genes of B. trypoxylicola into pBAD24

To clone *B. trypoxylicola motPS* genes into the downstream region of the arabinose-inducible promoter, pGEM-BtPS was digested with EcoRI and XbaI to cut out the *motPS* genes and ligated to *EcoR*I- and *Xba*I-digested pBAD24. The ligation mixture was transformed into *E. coli* DH5α MCR, and the transformed cells were applied to LB plates supplemented with ampicillin to a final concentration of 100 μg/mL and cultured at 37 °C overnight. The desired plasmid was obtained and named pBAD-BtPS.

### 2.4. Cloning of the Hybrid Stator Gene into the pGEM7zf (+) Vector and Integration Vector pAX01 for Bacillus Subtilis

*B. trypoxylicola*-derived Bt-MotP and Bt-MotS, *B. alcalophilus*-derived Ba-MotP and Ba-MotS and *B. pseudofirmus*-derived Bp-MotP and Bp-MotS were each replaced at the subunit level, and hybrid stators were constructed (Figure 1B).

First, each Bt-*motP*, Bt-*motS*, Ba-*motP*, Ba-*motS*, Bp-*motP,* and Bp-*motS* was separately amplified by PCR using each chromosomal DNA as a template. Each amplified gene product was ligated to each other using a 2nd PCR for the Gene SOEing method [35] to form a different combination of *motP* and *motS*. This popular method is based on PCR, recombines DNA sequences independently of restriction sites, and directly produces mutant DNA fragments in vitro. The combination of the primers used for PCR is shown in Figure 1B, and the information on the primers is shown in Table S1. Phusion High-Fidelity DNA Polymerase (New England Biolabs, Ipswich, MA, USA) was used for the PCR. The PCR composition and reaction conditions were performed in accordance with the Phusion High-Fidelity DNA Polymerase protocol. The PCR product was purified in the same manner. The cloning vector pGEM7zf (+) was digested with *Sma*I. The *Sma*I-digested pGEM7zf (+) and each PCR product were ligated in the same manner. The ligation reaction solution was transformed into *E. coli* DH5α MCR. The transformed cells were applied to S-Gal/LB agar (Sigma-Aldrich, St. Louis, MO, USA) supplemented with ampicillin to a final concentration of 100 μg/mL and cultured at 37 °C overnight. The desired plasmids were obtained, and each constructed plasmid was named pGEM-BtPS (Figure 1(B1)), pGEM-tpPS (Figure 1(B2)), pGEM-taPS (Figure 1(B3)), pGEM-ptPS (Figure 1(B5)), pGEM-paPS (Figure 1(B6)), pGEM-BaPS (Figure 1(B7)), pGEM-atPS (Figure 1(B8)), and pGEM-apPS (Figure 1(B9)). Sequence analysis confirmed that each obtained plasmid was free from mutations.

Under the control of the P*_xylA_* promoter, each *mot* gene constructed above was cloned into pAX01, a plasmid vector for integrating a foreign gene into the *lacA* region of the *B. subtilis* chromosome. pGEM-BtPS, pGEM-tpPS, pGEM-taPS, pGEM-ptPS, pGEM-paPS, and pAX01 were digested with *Sac*II. pGEM-BaPS, pGEM-atPS, pGEM-apPS, and pAX01 were digested with *Bam*HI and *Sac*II. The ligation solution was added to competent *E. coli* DH5α MCR cells in the same manner. The transformed cells were spread on an LB plate supplemented with ampicillin to a final concentration of 100 μg/mL and cultured at 37 °C overnight. The desired plasmids were obtained, and each constructed plasmid was named pAX-BtPS, pAX-tpPS, pAX-taPS, pAX-ptPS, pAX-paPS, pAX-BaPS, pAX-atPS, and pAX-atPS. pGEM-BpPS, and pAX-BpPS were not constructed because strain OF4PS had been constructed previously [23].

### 2.5. Construction of B. subtilis Integration Mutants Expressing the Hetero Hybrid Stator

Competent cells of the *B. subtilis* ΔABΔPS strain were prepared using the conventional method with Spizizen medium [36]. pAX-tpPS, pAX-ptPS, pAX-paPS, pAX-apPS, pAX-taPS, pAX-atPS pAX-BtPS, and pAX-BaPS were added to competent cells, and transformation was performed by the method of Spizizen et al. [36]. The transformed cells were spread onto S-Gal/LB agar plates supplemented with erythromycin to a final concentration of 1 μg/mL and cultured at 37 °C overnight. Transformants were selected from the colonies by color selection. Each positive colony was designated as strains TPPS, PTPS, PAPS, APPS, TAPS, ATPS, TTPS, and AAPS. Each transformant was cultured for 16 h at 37 °C and 200 rpm in LB supplemented with erythromycin to a final concentration of 1 μg/mL. Each genomic DNA sample was prepared from the culture using the UltraClean Microbial DNA Kit (QIAGEN, Hinden, Germany). The experiment was performed according to the manufacturer’s protocol. The genomic DNA of the TPPS, PTPS, PAPS, APPS, TAPS, ATPS, TTPS, and AAPS strains were used as templates, the stator gene region of each constructed strain was amplified by PCR with suitable primers. Phusion High-Fidelity DNA Polymerase was used for PCR. The PCR product was purified, and DNA sequence analysis was performed with the suitable primer set; it was confirmed that there was no mutation in the stator gene of the obtained integration mutants.

### 2.6. Growth Media and Growth Conditions for Growth and Swimming Assays

*B. trypoxylicola*, *B. alcalophilus*, and *B. pseudofirmus* were cultured in alkaline complex medium [21] (89 mM K_2_HPO_4_, 33 mM KH_2_PO_4_, 1 mM citric acid monohydrate, 0.4 mM MgSO_4_, 5% peptone, 2% yeast extract, 100 mM Na_2_CO_3_, 28 mM glucose) at 30 °C for 16 h with shaking. This was used as a preculture. Two milliliters of Tris medium [23] (30 mM Tris base, 7 mM citric acid monohydrate, 0.05% (w/v) yeast extract, 50 mM glucose and 1% (v/v) trace elements [37] pH 9.0) was inoculated so that the OD_600_ became 0.01, and the culture was grown at 30 °C for 16 h with shaking. The final OD _600_ of each culture was measured. Three independent experiments were conducted.

For *B. trypoxylicola*, *B. alcalophilus,* and *B. pseudofirmus* swimming speed assays in liquid, each strain was precultured in alkaline complex medium [21] (89 mM K_2_HPO_4_, 33 mM KH_2_PO_4_, 1 mM citric acid monohydrate, 0.4 mM MgSO_4_, 5% peptone, 2% yeast extract, 100 mM Na_2_CO_3_, 28 mM glucose) at 30 °C for 16 h with shaking. The culture was inoculated at an OD_600_ of 0.01 with fresh medium and cultured with shaking at 30 °C for approximately 7 to 8 h. The cultures were measured in 30 mM Tris-HCl containing 5 mM glucose and several NaCl or KCl concentrations at pH 9.0. The results represent the average swimming speed of 30 independent cells from three independent experiments. The error bars indicate the standard deviations.

To observe the effect of sodium and potassium ions on the swimming speed of *E. coli* mutant strains, the Bt-PS, Ba-PS, and ΔAB mutant strains were grown for 7 h at 30 °C in LB medium containing 0.1% arabinose with shaking. Cells were suspended in 1 mL of swimming buffer (pH 7.0) plus several different concentrations of NaCl or KCl and then incubated at 30 °C for 10 min. The swimming buffer contained 30 mM N-Tris(hydroxymethyl)methyl-2-aminoethanesulfonic acid (TES), 5 mM glucose, 0.1% arabinose, adjusted to pH 7.0 with *N*-methyl-D-glucamine. The results represent the average swimming speed of 30 independent cells from three independent experiments.

*E. coli* W3110 (wild-type) and Bt-PS were grown in liquid LB medium at 30 °C for 16 h with shaking. Each strain was inoculated into the same medium inoculated at an OD_600_ adjusted to 0.01 with fresh medium and cultured with shaking at 30 °C for approximately 6 to 7 h. If necessary, ampicillin was added to a final concentration of 0.1 mg/mL. For swimming speed assays of *B. pseudofirmus* OF4, strain OF4 was grown in liquid MYE medium (pH 10.5) with shaking at 30 °C for 7 h. Cells were suspended in 1 mL of swimming buffer (pH 7.0) plus several carbonyl cyanide m-chlorophenyl hydrazine (CCCP) concentrations, 5-(*N*-ethyl-*N*-isopropyl)-amiloride (EIPA) concentrations, or valinomycin concentrations and incubated at 30 °C for 10 min. The swimming buffer contained 30 mM TES, 5 mM glucose, 0.1% arabinose, 100 mM NaCl or 100 mM KCl, adjusted to pH 7.0 with *N*-methyl-D-glucamine. The results represent the average swimming speed of 30 independent cells from three independent experiments.

### 2.7. Swimming Assay of Mutant Strains Expressing Hybrid Stators 

For the measurement of swimming speed, *B. subtilis* (TPPS, PTPS, PAPS, APPS, TAPS, ATPS, TTPS, AAPS, and OF4PS) cells were aerobically grown on Spizizen I medium (30) (Spizizen salts, 0.5% glucose) supplemented with 1 μg/mL erythromycin, 10 μg/mL tryptophan and lysine at 37 °C for 16 h. The culture was inoculated into 20 mL of fresh medium supplemented with 1% (w/v) xylose at an OD_600_ of 0.01 and aerobically grown at 37 °C for approximately 6 to 7 h. Cells were suspended in 1 mL of swimming medium (pH 8.0) plus several NaCl concentrations plus KCl concentrations and were incubated at 37 °C for 10 min. The swimming medium contained 0.04% tryptone, 0.02% yeast extract, and 5 mM glucose, adjusted to pH 8.0 with *N*-methyl-D-glucamine. The results represent the average swimming speed of 30 independent cells from three independent experiments. Details of the swim analysis procedure are described in a separate section.

### 2.8. Cell Harvest Method for Swimming Assay and Swimming Video Recording Analysis

One hundred microliters of the culture broth were harvested by filtration on OMNIPORE membrane filters (0.45 µm), which were sandwiched between filter holders (SANSYO, Tokyo, Japan) connected to an aspirator MCV-20PS (ULVAC, Chigasaki, Japan), and suction was performed at 0.06 MPa. The filter was washed three times with 2 mL of the indicated buffer. The filter was placed in a 14 mL culture tube, suspended in 2 mL of the same buffer and incubated at 30 °C and 200 rpm for 10 min. Cell motility was observed under a dark-field microscope (Leica microscope DMLB100) and recorded in high definition with a digital color camera (model DFC310FX; Leica Microsystems, Tokyo, Japan). The swimming speed was determined with two-dimensional (2D) movement measurement capture 2 two-dimensional particle tracking velocimetry (2D-PTV) software (DigiMo, Tokyo, Japan). In addition, the swimming analysis was independently performed at least three times, the swimming speed of a total of 90 or more cells was measured, and the results were evaluated from the average value.

### 2.9. Measurement of Intracellular Potassium Concentration of E. coli HB-pBAD, TK-pBAD, TK-BaPS, and TK-BtPS

*E. coli* HB-pBAD, TK-pBAD, TK-BaPS and TK-BtPS cells were grown at 30 °C and 200 rpm for 16 h in LBK medium [30] (1% tryptone, 0.5% yeast extract, 83 mM KCl, pH 7.5) with 0.2% arabinose plus 100 μg/mL ampicillin. Then, each preculture was inoculated into modified TK2420 medium [38] (33.6 mM MOPS, 1 mM K_2_HPO_4_, 1.1 mM citric acid, 7.6 mM (NH_4_)_2_SO_4_, 6 mM FeSO_4_, 830 mM MgSO_4_, 10 mM glucose, 1 mg/mL thiamine and 0.2% arabinose, pH 7.0) with 0.2% arabinose, 100 μg/mL ampicillin plus 7 mM or 12 mM KCl, so that OD_600 nm_ was adjusted to 0.01. Growth was monitored hourly at OD_600 nm_. The cells were harvested by centrifugation (10,000 rpm (9100× *g*), 3 min, 25 °C) and washed with suspension in the same medium. Then, the cells were resuspended in 5 mL of 500 mM sucrose solution, centrifuged (10,000 rpm (9100× *g*), 3 min, 25 °C), and the supernatant was removed. This operation was repeated twice. Each cell protein was measured by the Lowry method using 100 μL of the cell suspension. The rest of the suspension was harvested and resuspended in 5 mL of 5% trichloroacetic acid (TCA) solution and shaken at 200 rpm at room temperature for 20 min. After shaking, centrifugation was performed at 10,000 rpm (9100× *g*) at 4 °C for 10 min, and 1 mL of the supernatant was collected. The supernatant was diluted 10 times and 100 times, and the K^+^ concentration was measured using a digital flame photometer ANA-135 (Tokyo Koden, Japan); a 50 ppm KCl solution was used as a standard. The intracellular K^+^ concentration (mM) was calculated using 1 mg of protein as a cell volume of 3 mL [30,39].

### 2.10. Phylogenetic Analysis and Multiple Alignment of the Transmembrane Domain Region of the B. trypoxylicola MotS Subunit

A phylogenetic analysis of the flagellar motor stator of the genus *Bacillus* was performed using DDBJ’s ClustalW (http://clustalw.ddbj.nig.ac.jp/index.php?lang=en) for alignment and the neighbor-joining method (NJ method) TreeView (http://code.google.com/p/treeviewx). The accession numbers of the strains used for analysis are as follows. *E. coli* MotB [EC_MotB (POAF06.1)], *B. clausii* KSM-K16 MotB [BC-MotB (BAD64519.9)], *B. subtilis* MotB [BS-MotB (CAB13241.1)], *B. subtilis* MotS [BS-MotS (CAB14950.1)], *B. alcalophilus* AV1934 MotS [BA-MotS (KGA96617.1)], *B. trypoxylicola* MotS [BT-MotS (CAB14950.1)], *B. pseudofirmus* OF4 MotS [BP-MotS (ADC48829.1)], *Bacillus halodurans* C-125 MotS [BH-MotS (BAB06958.1)], *Bacillus oceanisediminis* MotS [BO-MotS (WP_019383049)], *Bacillus cereus* MotB [BCS-MotB (KMN69571)], *Bacillus megaterium* MotB [BM-MotB (WP_013056745)], *Bacillus flexus* MotB [BF-MotB (WP_025909154)], and *Bacillus thermoamylovorans* MotB [BTH-MotB (WP_034770454)].

## 3. Results

### 3.1. Na^+^- or K^+^-Dependent Growth Capacities of Alkaliphiles, B. trypoxylicola, B. alcalophilus and B. pseudofirmus at pH 9.0

**Figure 2:**
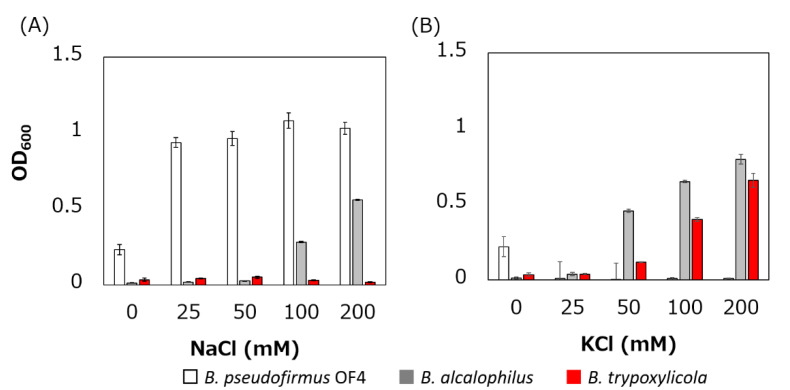
Effect of sodium and potassium ions on the growth of *B. trypoxylicola*, *B. alcalophilus,* and *B. pseudofirmus* OF4. Growth of bacterial cultures at 30 °C in Tris medium (pH 9) containing various concentrations of NaCl (**A**) or KCl (**B**) was monitored at OD_600_. The results are the averages of three independent experiments, and the error bars represent the standard deviations.

The growth of *B. trypoxylicola*, *B. alcalophilus* and *B. pseudofirmus* in Tris medium with several added Na^+^ and K^+^ concentrations was compared (Figure 2). As described previously, *B. alcalophilus* and *B. pseudofirmus* showed Na^+^- and K^+^-dependent and only Na^+^-dependent growth respectively in Tris medium at pH 9.0 [22,40]. *B. trypoxylicola* showed only K^+^-dependent growth. This result indicated that *B. trypoxylicola* prefers to use K^+^ rather than Na^+^ for growth in alkaline environments.

### 3.2. Swimming Assay of B. trypoxylicola, B. alcalophilus, and B. pseudofirmus under Various K^+^ and Na^+^ Concentrations

**Figure 3:**
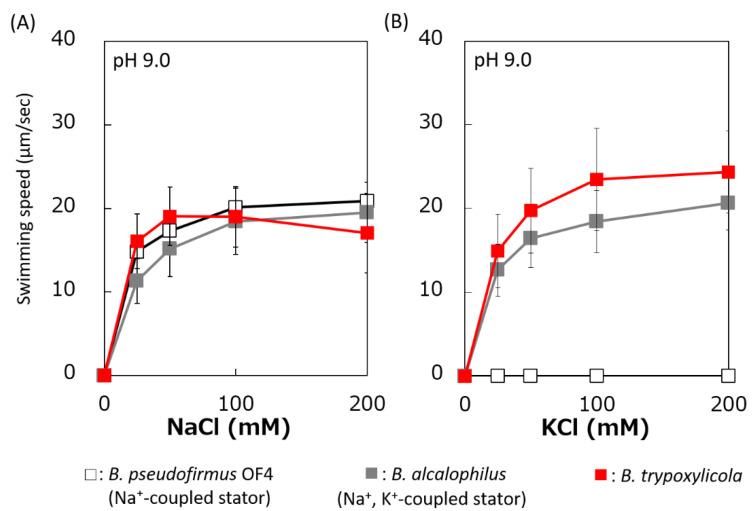
Effect of sodium and potassium ions on the swimming speed of *B. trypoxylicola*, *B. alcalophilus,* and *B. pseudofirmus* OF4. Swimming speeds of *B. trypoxylicola*, *B. alcalophilus*, and *B. pseudofirmus* OF4 cells were measured in 30 mM Tris-HCl containing 5 mM glucose and several NaCl (**A**) or KCl (**B**) concentrations at pH 9.0. The results represent the average swimming speed of 30 independent cells from three independent experiments. The error bars indicate the standard deviations.

The swimming speeds of *B. trypoxylicola*, *B. alcalophilus*, and *B. pseudofirmus* in swimming assay buffer with different K^+^ and Na^+^ concentrations were compared (Figure 3). Similar to previously reported results, *B. alcalophilus* swimming showed NaCl and KCl concentration dependence, and *B. pseudofirmus* swimming showed NaCl concentration dependence [22,40]. *B. trypoxylicola* swimming was observed under the same concentrations of NaCl and KCl as those used for *B. alcalophilus*. To summarize these results, it was suggested that *B. trypoxylicola* has a flagellar motor coupled to both K^+^ and Na^+^.

### 3.3. Identification of MotP/MotS Operon of B. trypoxylicola

**Figure 4:**
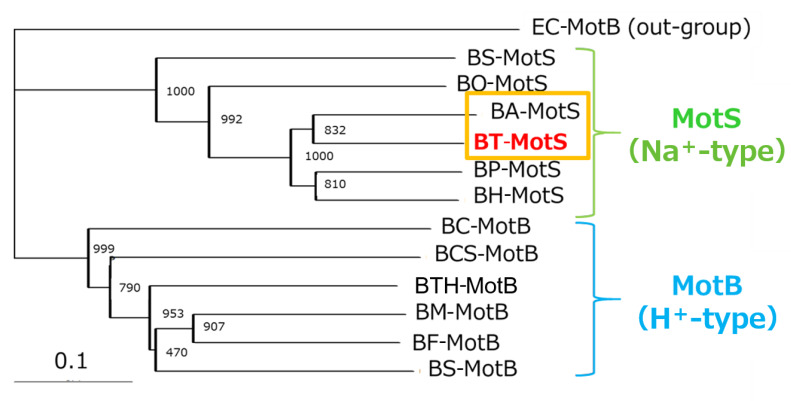
Phylogenetic tree of the stator subunits MotB and MotS of the flagellar motor from *Bacillus* spp. using the NJ method. A phylogenetic analysis of the flagellar motor stator of the genus *Bacillus* was performed. BT-MotS is shown in red. The Na^+^- and K^+^-coupled stator subunits, BT-MotS and BA-MotS are enclosed in ocher. The Na^+^-driven stator is surrounded by yellow-green, and the H^+^-driven stator is surrounded by light blue. The details are described in the Materials and Methods section. The scale bar indicates the number of amino acid substitutions per site. The numbers between the branches indicate the bootstrap values. Accession numbers for each protein are described in the Materials and Methods section. EC: *E. coli*, BS: *B. subtilis*, BO: *B. oceanisediminis*, BA: *B. alcalophilus*, BT: *B. trypoxylicola*, BP: *B. pseudofirmus*, BH: *B. halodurans*, BC: *B. clausii*, BCS: *B. cereus*, BTH: *B. thermoamylovorans*, BM: *B. megaterium*, and BF: *B. flexus*.

**Figure 5:**
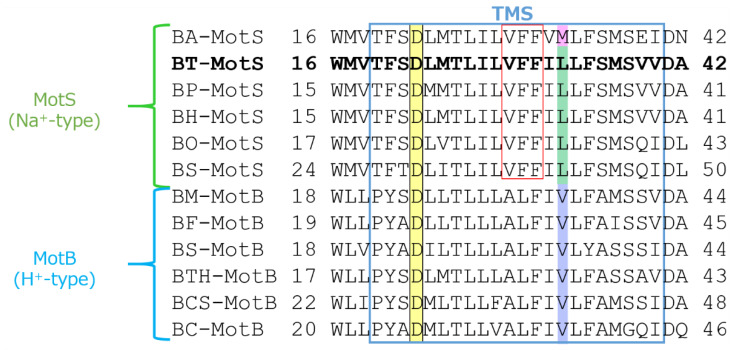
Multiple alignment of the regions containing the single transmembrane segment of MotB-type stator proteins from *Bacillus* spp. using ClustalW. Alignment of the flagellar motor stator of *Bacillus* was performed. The single transmembrane segment (TMS) is enclosed in blue. The sequence of BT-MotS is shown in bold. Aspartic acid residues at the putative coupling ion-binding site are highlighted yellow. A leucine residue is conserved in MotS and a valine residue is conserved in MotB and are highlighted green and purple, respectively. In BA-MotS, the methionine residue is highlighted pink. Na^+^ and amiloride binding motif (VFF) are enclosed in red. Accession numbers for each protein are described in the Materials and Methods section. EC: *Escherichia coli*, BS: *B. subtilis*, BO: *B. oceanisediminis*, BA: *B. alcalophilus*, BT: *B. trypoxylicola*, BP: *B. pseudofirmus*, BH: *B. halodurans*, BC: *B. clausii*, BCS: *B. cereus*, BTH: *B*. *thermoamylovorans*, BM: *B. megaterium*, and BF: *B. flexus*.

The draft genome of *B. trypoxylicola* NBRC102646 was retrieved from the DDBJ/EMBL/GenBank databases (accession number BCWA00000000.1). The annotation of the draft genome sequence shows that *B. trypoxylicola* has a *motP*/*motS* operon: Bt-*motP*/Bt-*motS*. The GenBank accession numbers and numbers of amino acids in Bt-MotP and Bt-MotS are WP_045481010.1 and 267 aa and WP_061950140.1 and 247 aa, respectively. The amino acid sequence was compared with other stator sequences of *Bacillus* spp. using ClustalW. Bt-MotS was classified as a MotP/MotS family (Figure 4). Bt-MotP and Bt-MotS were closely related to *B. alcalophilus* MotP (Ba-MotP) and MotS (Ba-MotS) (74% and 73% identity and 87% and 88% similarity, respectively). In addition, in the transmembrane region of the MotB and MotS subunits of *Bacillus* spp., the aspartic residue, which is a presumed coupling cation-binding site, is entirely conserved (Figure 5) [26]. It is known that valine residues are highly conserved in the H^+^-coupled MotB subunit and that leucine residues are highly conserved in the Na^+^-coupled MotS subunit ten amino acids downstream of this aspartic acid. In Bt-MotS, the leucine residue was preserved at that position. These results indicated that Bt-MotP/Bt-MotS is a stator belonging to the MotP/MotS family.

### 3.4. Swimming Assay of an E. coli Stator-Deficient Strain Expressing Bt-MotP/Bt-MotS

**Figure 6:**
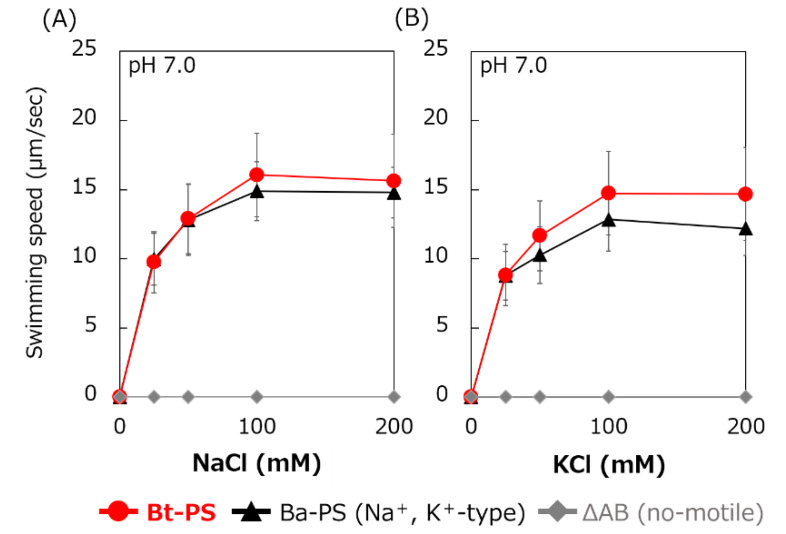
Effect of sodium and potassium ions on the swimming speed of *E. coli* mutant strains. The Bt-PS, Ba-PS, and ΔAB mutant strains were grown for 7 h at 30 °C in LB medium containing 0.1% arabinose with shaking. Cells were suspended in 1 mL of swimming buffer (pH 7.0) plus several NaCl concentrations (**A**) and several KCl concentrations (**B**) and were incubated at 30 °C for 10 min. Swimming buffer contained 30 mM TES, 5 mM glucose, and 0.1% arabinose, adjusted to pH 7.0 with *N*-methyl-D-glucamine. The results represent the average swimming speed of 30 independent cells from three independent experiments. The error bars indicate the standard deviations.

It is unknown whether Bt-MotP/Bt-MotS uses Na^+^ and K^+^ as coupling ions. Therefore, the following experiment was carried out to confirm whether Bt-MotP/Bt-MotS uses these cations. An *E. coli* stator-deficient strain (ΔAB) expressing Bt-MotP/Bt-MotS (strain Bt-PS) was constructed. The swimming speeds of strain Bt-PS, strain ΔAB expressing Ba-MotP/Ba-MotS (strain Ba-PS) and strain ΔAB carrying pBAD24 in TES buffer solution (pH 7.0) were compared at several concentrations of KCl or NaCl (Figure 6). No motility was observed in the ΔAB strain carrying pBAD24 at any KCl or NaCl concentration. The Bt-PS and Ba-PS strains showed no motility in the absence of KCl or NaCl but showed KCl or NaCl concentration-dependent motility. These results suggested that Bt-MotP/Bt-MotS utilizes both Na^+^ and K^+^ as coupling cations.

### 3.5. Growth of an E. coli K^+^ uptake System-Deficient Strain Expressing Bt-MotP/Bt-MotS and Measurement of the Intracellular K^+^ Concentration

**Figure 7:**
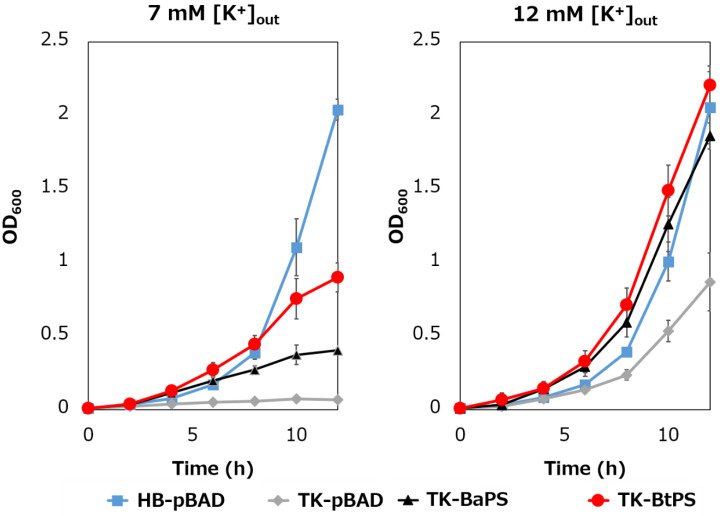
Effect of extracellular K^+^ concentrations on the growth of *E. coli* and its K^+^ uptake system-deleted mutant strains. The HB-pBAD, TK-pBAD, TK-BaPS, and TK-BtPS strains were grown for 12 h at 30 °C in a modified TK2420 medium (pH 7.0) contained 7 mM or 12 mM KCl with 0.2% arabinose with shaking. Growth was assessed hourly at OD_600_.

**Figure 8:**
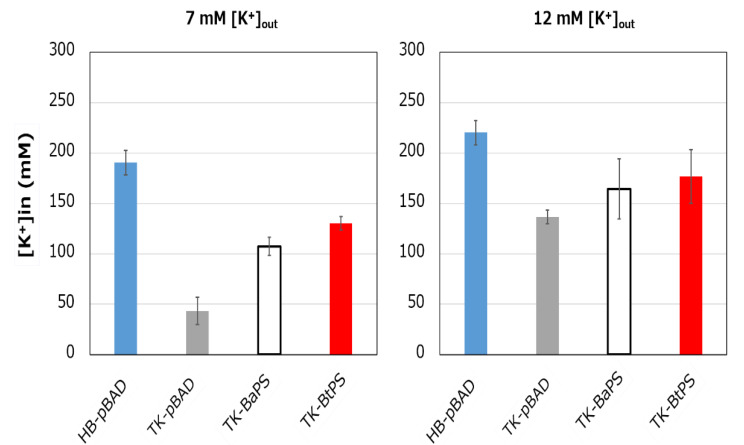
Effects of extracellular K^+^ concentrations on the intracellular K^+^ concentration of *E. coli* and its K^+^ uptake system-deleted mutant strains. The HB-pBAD, TK-pBAD24, TK-BaPS, and TK-BtPS strains were grown for 12 h at 30 °C in modified TK2420 medium (pH 7.0) contained 7 mM or 12 mM KCl with 0.2% arabinose with shaking. K^+^ concentrations per cell were measured as described in the Materials and Methods section.

It was unknown whether the cells uptake K^+^ via the stator complex Bt-MotP/Bt- MotS. Therefore, to confirm this, the following experiment was conducted. The major K^+^ uptake system-deficient strain *E. coli* TK2420 cannot grow in medium containing ≤ 10 mM K^+^; however, *E. coli* HB101 (wild-type) is capable of growing under this condition. Therefore, strain TK2420 expressing Bt-MotP/Bt-MotS (strain TK-BtPS) was constructed, and a growth experiment and intracellular K^+^ concentration measurements were conducted under the conditions of 7 mM and 12 mM K^+^ or less. Similarly, growth experiments and intracellular K^+^ concentration measurements of strains HB101/pBAD24 (strain HB-pBAD), TK2420/pBAPS (strain TK-BaPS), and TK2420/pBAD24 (strain TK-pBAD) were also conducted (Figure 7 and Figure 8). As a result, under the 7 mM K^+^ growth condition, strain HB-pBAD showed good growth even though a growth lag was observed during the early stage of culture. Under similar conditions, two strains (TK-BaPS and TK-BtPS) grew, but growth was worse than that of strain HB-pBAD. On the other hand, TK-pBAD showed no growth. Under the 12 mM K^+^ growth condition, all strains showed the same growth except strain TK-pBAD, which showed the slowest growth. Furthermore, under the 7 mM K^+^ growth condition, strains HB-pBAD, TK-BaPS, and TK-BtPS clearly showed higher intracellular K^+^ concentrations than strain TK-pBAD. Under the 12 mM K^+^ growth condition, the intracellular K^+^ concentration in all strains was more than 140 mM. These results suggest that Bt-MotP/Bt-MotS can take up K^+^ into cells.

### 3.6. Swimming Assay of E. coli Stator-Deficient Strain Expressing Bt-MotP/Bt-MotS With/Without a Flagellar Motor Inhibitor

**Figure 9:**
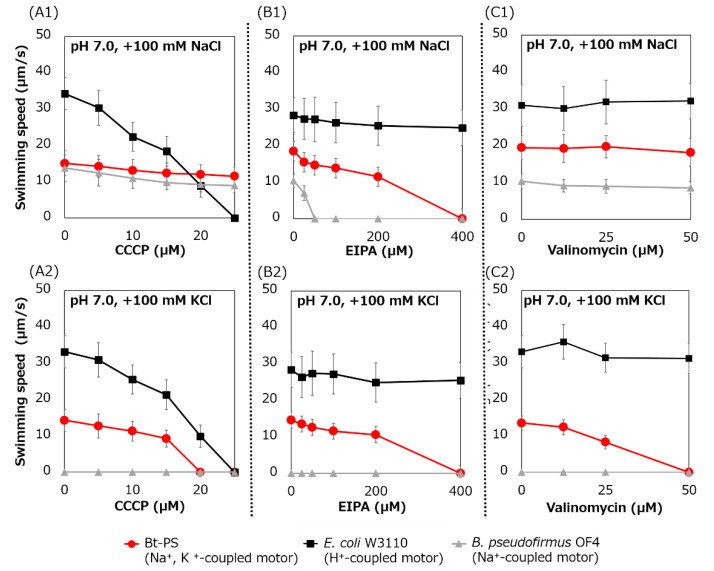
Swimming assay of BTPS (a stator-less *E. coli* mutant expressing Bt-MotPS) in the presence of flagellar motor inhibitor. The Bt-PS and *E. coli* W3110 strains were grown for 7 h at 30 °C in LB medium containing 0.1% arabinose with shaking. *B. pseudofirmus* OF4 was grown for 7 h at 30 °C in MYE medium (pH 10.5) with shaking. Cells were suspended in 1 mL of swimming buffer (pH 7.0) plus several carbonyl cyanide m-chlorophenyl hydrazine (CCCP) concentrations (**A1** and **A2**), plus several 5-(*N*-ethyl-*N*-isopropyl)-amiloride (EIPA) concentrations (**B1** and **B2**), plus several valinomycin concentrations (**C1** and **C2**) and were incubated at 30 °C for 10 min. Swimming buffer contained 30 mM TES, 5 mM glucose, 0.1% arabinose, 100 mM NaCl (**A1**, **B1** and **C1**) or 100 mM KCl (**A2**, **B2** and **C2**), adjusted to pH 7.0 with *N*-methyl-D-glucamine. The results represent the average swimming speed of 30 independent cells from three independent experiments. The error bars indicate the standard deviations.

Swimming inhibition assays of strains Bt-PS, *E. coli* W3110, and *B. pseudofirmus* were performed using CCCP, EIPA, and valinomycin. CCCP is a protonophore and an inhibitor of the H^+^-coupled stator [41], EIPA is an amiloride analog and an inhibitor of the Na^+^-coupled stator [15,25] and valinomycin is an ionophore of K^+^ (Figure 9).

The swimming speed of strain Bt-PS decreased in response to increasing CCCP concentrations in only the presence of K^+^, and swimming was completely stopped at 20 μM CCCP (Figure 9(A1,A2)). On the other hand, when Na^+^ was present in the buffer, the Bt-PS strain was observed to swim. Since *E. coli* W3110 has an H^+^-coupled motor, the swimming of *E. coli* W3110 was inhibited by elevated CCCP concentration under both KCl and NaCl conditions, and the swimming completely stopped at 25 μM CCCP. Since *B. pseudofirmus* has a Na^+^-coupled motor, the swimming of *B. pseudofirmus* was not inhibited by CCCP in the presence of NaCl. The ion motive force (IMF) is composed of the cell membrane voltage (*V*_m_) and the ion concentration gradient of inside and outside the cell. In general, *E. coli* cells maintain a lower Na^+^ concentration and a higher K^+^ concentration inside the cell compared to the outside [42,43]. K^+^ concentration gradient (ΔpK) is smaller than Na^+^ concentration gradient (ΔpNa). Therefore, the potassium motive force (potassium MF) has a higher dependence on the *V*_m_ than the sodium motive force (SMF). The inhibition of Bt-PS swimming in the presence of KCl may be due to the reduction of the *V*_m_ of the potassium MF by CCCP.

The swimming speed of the Bt-PS strain decreased in response to elevated EIPA concentrations under both KCl and NaCl conditions, and swimming completely stopped at 400 μM EIPA (Figure 9(B1,B2)). Since *E. coli* W3110 has an H^+^-coupled motor, the swimming of *E. coli* W3110 was not inhibited by EIPA under both KCl and NaCl conditions. Since *B. pseudofirmus* has a Na^+^-coupled motor, the swimming of *B. pseudofirmus* was inhibited by elevated EIPA concentrations in the presence of NaCl. The reason that the strain Bt-PS was sensitive to EIPA under both conditions is presumed to be that EIPA binds to the coupling ion transport pathway of the stator complex. Kuroda et al. reported that VFF sequence are present in many Na^+^ coupled transport proteins and proposed that VFF sequence is a motif involved in Na^+^ binding and amiloride binding [44]. A VFF sequence can be found in the transmembrane region of the Na^+^-coupled MotS subunit (Figure 5). There are multiple reports that the EIPA and its analog phenamil inhibited the coupling ion transport pathway in the stator complex [17,45,46,47,48]. Previously, a similar inhibition experiment was carried out using Ba-MotP/Ba-MotS of *B. alcalophilus*, which can utilize both Na^+^ and K^+^ as coupling ions, and EIPA was inhibited under both K^+^ and Na^+^ conditions as in strain Bt-PS [22].

The swimming speed of strain Bt-PS decreased in response to elevated valinomycin concentrations in only the presence of K^+^, and swimming completely stopped at 50 μM valinomycin (Figure 9(C1,C2)). On the other hand, the swimming of Bt-PS was not inhibited by valinomycin in the presence of NaCl. Since *E. coli* W3110 has an H^+^-coupled motor and *B. pseudofirmus* has a Na^+^-coupled motor, *E. coli* W3110 and *B. pseudofirmus* swimming was not inhibited by valinomycin. The reason that Bt-PS swimming was inhibited by valinomycin in the presence of KCl was hypothesized to be the decrease in the potassium MF to rotate the flagella due to the valinomycin-induced loss of the ΔpK.

### 3.7. Functional Analysis of the Hybrid Stator With the Na^+^-Coupled MotP/MotS Subunit Replaced With the Na^+-^ and K+-Coupled MotP/MotS Subunit

**Figure 10:**
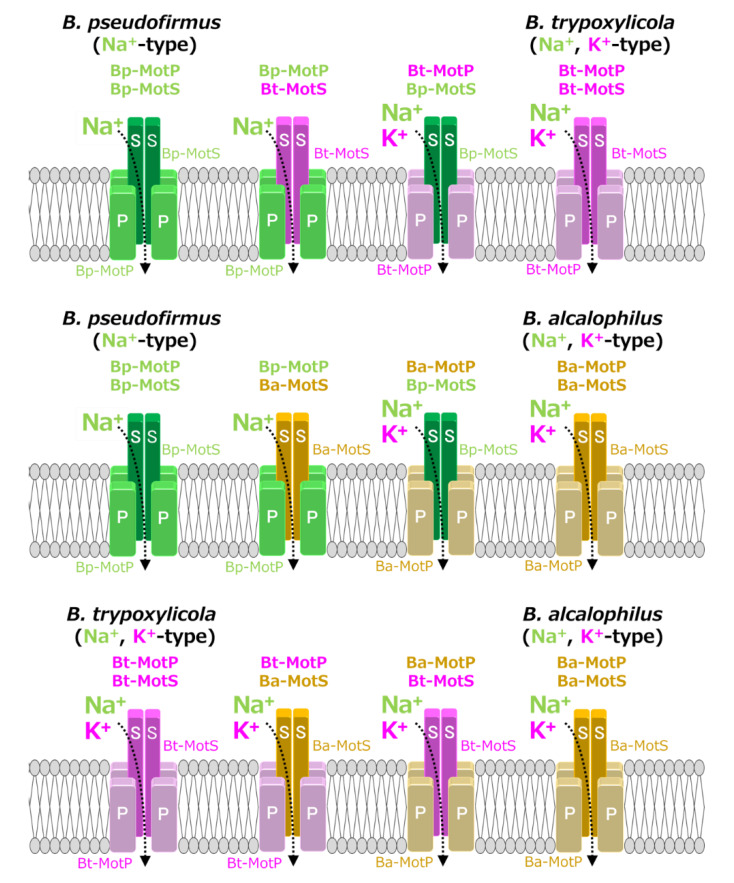
Schematic diagram of the stators of wild-type MotP/MotS and hybrid MotP/MotS. The coupling ions of each hybrid stator shown here are based on the results in Figure 11.

**Figure 11:**
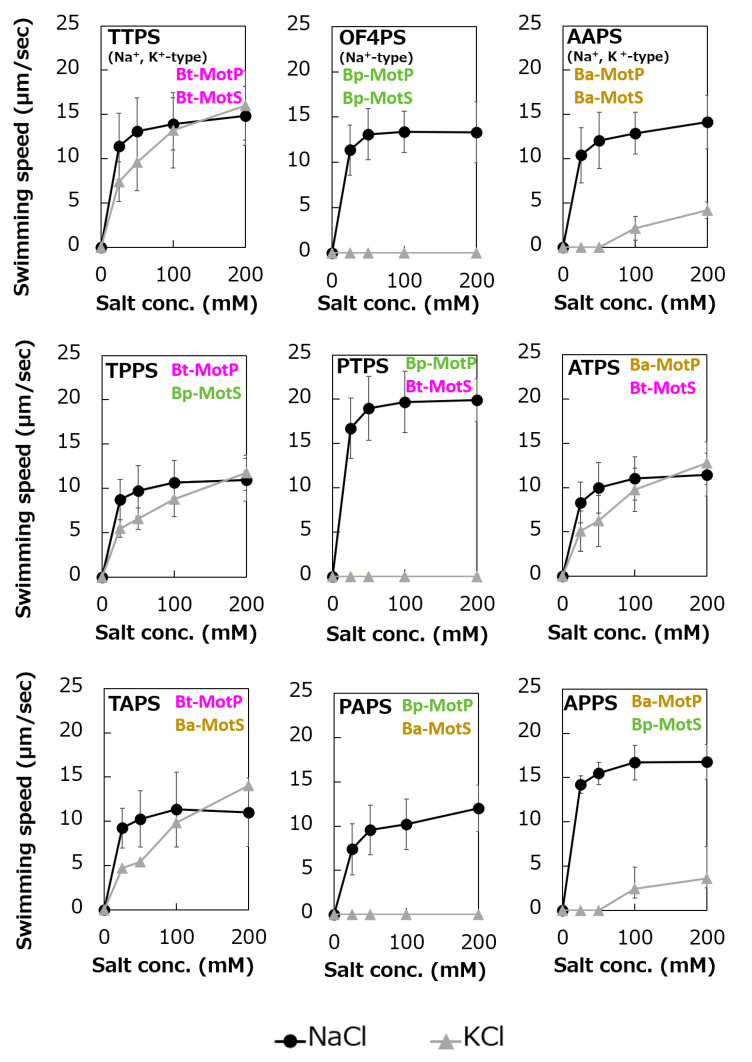
Swimming assays of hybrid stators between Na^+^-type stators and Na^+^ and K^+^-type stators. *B. subtilis* strains were grown for 6 h at 37 °C in Spizizen I medium 1% xylose with shaking. Cells were suspended in 1 mL of swimming medium (pH 8.0) plus several NaCl concentrations plus several KCl concentrations and then incubated at 37 °C for 10 min. Swimming medium contained 0.04% tryptone, 0.02% yeast extract, and 5 mM glucose, adjusted to pH 8.0 with *N*-methyl-D-glucamine. The results represent the average swimming speed of 30 independent cells from three independent experiments. The error bars indicate the standard deviations.

MotB-type (MotB, MotS, and PomB) subunits are thought to be particularly important for coupling cation selectivity of the stator, but details of the mechanism are still unknown. The data of Figure 6 and Figure 9 suggested that the stator of *B. trypoxylicola* was both the Na^+^- and K^+^-coupled stators MotP and MotS. A leucine residue was the important amino acid for ion selectivity in the transmembrane region of the Bt-MotS subunit (Figure 5) [21]. Furthermore, the amino acid sequences in the transmembrane region between the Bt-MotS subunit and the Na^+^-coupled MotS subunit of *B. halodurans* C-125 (Bh-MotS) were 100% identical (Figure 5). This suggests that the Bt-MotP subunit is important for the mechanism of K^+^ selectivity of the *B. trypoxylicola* stator Bt-MotP/Bt-MotS. Therefore, we focused on the exchange of the Na^+-^ and K^+^-coupled MotP and MotS subunits and the Na^+^-coupled MotP and MotS subunits. The hybrid subunits were constructed using *B. trypoxylicola*-derived Na^+^- and K^+^-coupled Bt-MotP and Bt-MotS, *B. alcalophilus*-derived Na^+-^ and K^+^-coupled Ba-MotP and Ba-MotS, and *B. pseudofirmus*-derived Na^+^-coupled Bp-MotP and Bp-MotS. The constructed hybrid combinations are shown in Figure 10. The coupling ions of each hybrid stator shown in Figure 9 are based on the results in Figure 11.

To analyze the ion selectivity of the constructed hybrid stators, swimming analysis was performed using mutant strains expressing the hybrid stator constructed in the *B. subtilis* stator-deficient strain (Figure 11).

Strains OF4PS, PTPS, and PAPS showed Na^+^-coupled swimming behavior. However, there was no K^+^-dependent motility among them. These results suggest that the hybrid stators Bp-MotP/Bt-MotS and Bp-MotP/Ba-MotS are Na^+^-driven stators that can use Na^+^ as a coupling ion. These hybrid stators did not exhibit K^+^ concentration-dependent motility while having Na^+^- and K^+^-coupled MotS (Bt-MotS from *B. trypoxylicola* or Ba-MotS from *B. alcalophilus*) subunits. Thus, the K^+^ selectivity of the stator indicated that the MotP subunit is critical, but the MotS subunit is not. On the other hand, strains AAPS, which had *B. alcalophilus* MotP/MotS; and TTPS, which had a *B. trypoxylicola* MotP/MotS stator; and strains TPPS, APPS, TAPS, and ATPS with hybrid stators showed both Na^+^- and K^+^-coupled motility. From these results, the hybrid stators Bt-MotP/Bp-MotS, Ba-MotP/Bp-MotS, Bt-MotP/Ba-MotS, and Ba-MotP/Bt-MotS coupled with Na^+^ and K^+^ as their coupling cations. These strains commonly have Na^+^- and K^+^-coupled MotP subunits. Therefore, it was suggested that the MotP subunit that forms an ion channel together with the MotS subunit is more important for the K^+^ selectivity of the flagellar motor stator than the MotS subunit itself.

## 4. Discussion

*B. trypoxylicola*, a species closely related to *B. alcalophilus*, requires K^+^ for growth, suggesting that *B. trypoxylicola* has a homeostatic ability to utilize K^+^ [22]. Generally, alkaliphilic bacteria isolated from soil require Na^+^ for growth, whereas alkaliphilic bacteria isolated from human feces and the guts of insects require K^+^ in addition to Na^+^ for growth [3,49,50,51]. Gut portions of soil-feeding termites generally contain large amounts of potassium ions, and their pH is extremely alkaline [52,53]. Gut alkalinity helps solubilize and facilitate the uptake of soil organic matter [54]. Potassium-requiring alkaliphilic bacteria have been isolated from such environments [3,49,50]. *B. trypoxylicola* was isolated from the gut of the Japanese beetle [27]. This suggests that *B. trypoxylicola* has adaptively evolved into the K^+^-rich gut environment for growth and motility. *B. trypoxylicola* was the second example of a flagellar motor that could use Na^+^ and K^+^ as coupling ions. Most of the MotP/MotS stators derived from alkaliphilic *Bacillus* spp. can function only with Na^+^ [16,55]. The flagellar motor of *B. trypoxylicola* is the second example of a MotP/MotS stator that can use both Na^+^ and K^+^. Even though *B. trypoxylicola* exhibits no requirement of Na^+^ for growth, the flagellar motor can utilize both Na^+^ and K^+^. This suggests that the ancestor of the MotP/MotS stator is originally a Na^+^ type and that the MotP/MotS stator of *B. trypoxylicola* acquired the ability to use K^+^ as a coupling ion in addition to Na^+^ during the evolutionary process in a K^+^-rich environment.

Previous studies have suggested that the transmembrane domain of the MotB-type (MotB, MotS, and PomB) subunit is particularly critical for the coupling ion selectivity of Na^+^ and H^+^ in the flagellar motor stator [21,25]. With respect to K^+^ selectivity, MotS-M33 was reported to be important in the flagellar motor of *B. alcalophilus* [22]. However, a methionine residue is not conserved at a similar site in BT-MotS, and general MotS types conserve a leucine residue at this site. It is suggested that this site is not necessarily essential for K^+^ selectivity. Furthermore, the amino acid sequence in the transmembrane segment of the Bt-MotS subunit was identical to that of the MotS subunit (Bh-MotS) of Na^+^-driven MotP/MotS of *B. halodurans* C-125 (Figure 5). Therefore, it was concluded that the MotP subunit that forms an ion channel with MotS is important for the K^+^ selectivity of the flagellar stator.

The swimming assay with various inhibitors against strain Bt-PS, strain Bt-PS showed sensitivity to CCCP, EIPA, and valinomycin in the presence of K^+^. On the other hand, in the presence of Na^+^, sensitivity was observed for only EIPA (Figure 9). Strain Bt-PS was sensitive to the H^+^-coupled flagellar inhibitor CCCP in the presence of K^+^. This indicates that the *V*_m_ of the potassium MF is dominant for rotation of the flagellar motor. The results for these inhibitors showed that the flagellar motor, which can utilize both K^+^ and Na^+^, contributes a great deal to the *V*_m_ when using the potassium MF. It was also shown that when using the SMF, the contribution of the ΔpNa is greater than that of the *V*_m_. In addition, the results show for the first time that valinomycin is useful as an inhibitor of the K^+^-coupled flagellar motor.

According to experiments using the hybrid stators MotA/MotS and MotP/MotB that replace the H^+^-coupled stators MotA/MotB and Na^+^-coupled stators MotP/MotS of *B. subtilis*, the MotB subunit defines H^+^ selectivity, and the MotS subunit defines Na^+^ selectivity [25]. In summary, when H^+^ or Na^+^ is selected as the coupling ion for ion selectivity of the flagellar motor stator of bacteria from the genus *Bacillus* and its closely related species, the transmembrane region of the MotB or MotS subunit is considered to be particularly important. On the other hand, this study suggested for the first time that the MotP subunit is important when selecting K^+^ as a coupling ion. In the MotP subunit of *B. subtilis*, the structure of the pathway of the coupling ion is changed by the mutation of MotP-L170P of the third transmembrane region, and the motility of the MotS-D30E mutant is improved [21]. In this way, the MotP subunit may influence the ion selectivity of coupling ions. It is speculated that an ancestral type of Bt-MotP/Bt-MotS was originally considered the Na^+^-coupled stator MotP/MotS, and in the process of adapting to a K^+^-rich environment, the Bt-MotP subunit was mutated, and the structure of the coupling ion pathway evolved to use not only Na^+^ but also K^+^.

**Figure 12:**
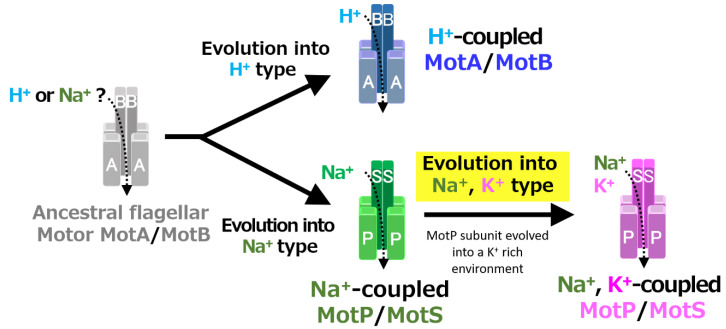
Evolutionary hypothesis of bacterial flagellar stator based on the results. It has not been clarified which ancestral flagellar motor stator utilized H^+^ or Na^+^ as the coupling ion of the flagellar motor. It is assumed that the ancestral stator evolved into H^+^-coupled and Na^+^-coupled stators. The MotB-type subunit is critical for the coupling ion selectivity of H^+^ and Na^+^. Furthermore, it is assumed that the Na^+^-coupled stator evolved into the Na^+^- and K^+^-coupled stator. The MotP subunit is critical for the coupling ion selectivity of K^+^. Therefore, *B. alcalophilus* and *B. trypoxylicola* are assumed to have evolved these properties to adapt to a potassium-rich environment.

A recent study reported that *Aquifex aeolicus*, a hyperthermophilic bacterium thought to have branched off during the early stages of bacterial evolution, has a flagellar motor that utilized Na^+^ as a coupling ion, and it has been proposed that the motor of the bacterial ancestor may have used Na^+^ as a coupling ion [56]. It is believed that the initial flagellum motor adaptively evolved to an H^+^-coupled stator and an Na^+^-coupled stator, respectively, in accordance with a growing environment. A primitive stator is estimated to have evolved by a motor utilizing only H^+^ or Na^+^ by mutating the amino acid residues of the MotB-type subunit. After that, it is estimated that the Na^+^-coupled stator evolved into a Na^+^- and K^+^-coupled stator to adapt to an environment (such as in the guts of living organisms) where K^+^ exists abundantly. These processes are outlined in Figure 12.

In the future, the details of the K^+^ selection mechanism of the flagellar motor stator should be clarified by focusing on the transmembrane regions of the MotP subunit.

## 5. Conclusions

Previously, the coupling ion selection of H^+^ or Na^+^ for flagellar motor stators of *Bacillus* spp. and its relatives suggested that the transmembrane region of the MotB or MotS subunit is particularly critical. This study suggests for the first time that the MotP subunit is critical when selecting K^+^ as the coupling ion. The K^+^- and Na^+^-coupled MotP/MotS stator complexes from alkaliphilic *B. alcalophilus* and *B. trypoxylicola* were presumed to have evolved from Na^+^-coupled MotP/MotS stator complexes during adaptation to a large potassium-rich environment. This work could provide a new perspective on the study of the ion selectivity of flagellar motor stators.

## Figures and Tables

**Table 1:** The bacterial strains used in this study.

Strain	Description	Source or Reference
*Bacillus trypoxylicola*	wild type (NBRC 102646)	[27]
*Bacillus alcalophilus*	wild type (JCM5652)	[28]
*Bacillus pseudofirmus* OF4	wild type	[29]
*Escherichia coli* strains		
W3110	F^-^ *IN (rrnD-rrnE)1*	R. Aono
DH5αMCR	F^-^ *mcrAΔ1 (mrr-hsd RMS-mcrBC) Φ80dlacZ Δ(lacZYAargF) U169 deoR recA1 endA1 supE44 λthi-1 gyr-496 relA1*	Stratagene
RP6665	Δ*motAB*	J. S. Parkinson
Bt-PS	RP6665, pBAD24 + *motPS* from *B. trypoxylicola*	This study
Ba-PS	RP6665, pBAD24 + *motPS* from *B. alcalophilus*	This study
TK2420	F^-^ *thi rha lacZ nagA Δ(kdpFAB) Δ(trk-mscL) trkD1*	[30]
TK-BtPS	TK2420, pBAD24 + *motPS* from *B. trypoxylicola*	This study
TK-BaPS	TK2420, pBAD24 + *motPS* from *B. alcalophilus*	[22]
TK-pBAD	TK2420, pBAD24	[22]
HB101	*supE44, Δ(mcrC-mrr), recA13, ara-14, proA2, lacY1, galK2, rpsL20, xyl-5, mtl-1, leuB6, thi-1*	Takara Bio
HB-pBAD	HB101, pBAD24	[22]
*Bacillus subtilis* strains		
BR151MA	*lys3 trpC2* (wild type)	[25]
ΔABΔPS	BR151MA Δ*motAB* Δ*motPS*	[31]
TTPS	ΔABΔPS *lacA*::P*_xylA_*-*motPS* from *B. trypoxylicola*	This study
OF4PS	ΔABΔPS *lacA*::P*_xylA_*-*motPS* from *B. pseudofirmus*	[23]
AAPS	ΔABΔPS *lacA*::P*_xylA_*-*motPS* from *B. alcalophilus*	This study
TPPS	ΔABΔPS *lacA*::P*_xylA_*-TP*-motPS* (*motP* from *B. trypoxylicola*, *motS* from *B. pseudofirmus*)	This study
PTPS	ΔABΔPS *lacA*::P*_xylA_*-PT*-motPS* (*motP* from *B. pseudofirmus*, *motS* from *B. trypoxylicola*)	This study
PAPS	ΔABΔPS *lacA*::P*_xylA_*-PA*-motPS* (*motP* from *B. pseudofirmus*, *motS* from *B. alcalophilus*)	This study
APPS	ΔABΔPS *lacA*::P*_xylA_*-AP*-motPS* (*motP* from *B. alcalophilus*, *motS* from *B. pseudofirmus*)	This study
TAPS	ΔABΔPS *lacA*::P*_xylA_*-TA*-motPS* (*motP* from *B. trypoxylicola*, *motS* from *B. alcalophilus*)	This study
ATPS	ΔABΔPS *lacA*::P*_xylA_*-AT*-motPS* (*motP* from *B. alcalophilus*, *motS* from *B. trypoxylicola*)	This study

**Table 2:** The plasmids used in this study.

Plasmid	Description	Source or Reference
pGEM7zf (+)	Cloning vector; Ap^R^	Promega
pGEM-T Easy	TA-cloning vector; Ap^R^	Promega
pBAD24	Expression vector; Ap^R^; P_BAD_ promoter	[32]
pAX01	*lacA* integration vector with Em^R^ gene and P*_xylA_* promoter upstream of multiple cloning site	[33]
pGEM-T-BtPS	pGEM-T Easy + *motPS* from *B. trypoxylicola*	This study
pGEM-BtPS	pGEM7zf (+) + *motPS* from *B. trypoxylicola*	This study
pGEM-BpPS	pGEM7zf (+) + *motPS* from *B. pseudofirmus*	This study
pGEM-BaPS	pGEM7zf (+) + *motPS* from *B. alcalophilus*	This study
pGEM-tpPS	pGEM7zf (+) +tp*-motPS* (*motP* from *B. trypoxylicola*, *motS* from *B. pseudofirmus*)	This study
pGEM-taPS	pGEM7zf (+) +ta*-motPS* (*motP* from *B. trypoxylicola*, *motS* from *B. alcalophilus*)	This study
pGEM-ptPS	pGEM7zf (+) +pt*-motPS* (*motP* from *B. pseudofirmus*, *motS* from *B. trypoxylicola*)	This study
pGEM-paPS	pGEM7zf (+) +pt*-motPS* (*motP* from *B. pseudofirmus*, *motS* from *B. alcalophilus*)	This study
pGEM-atPS	pGEM7zf (+) +at*-motPS* (*motP* from *B. alcalophilus*, *motS* from *B. trypoxylicola*)	This study
pGEM-apPS	pGEM7zf (+) +at*-motPS* (*motP* from *B. alcalophilus*, *motS* from *B. pseudofirmus*)	This study
pBAD-BtPS	pBAD24 + *motPS* from *B. trypoxylicola*	This study
pBAD-BaPS	pBAD24 + *motPS* from *B. alcalophilus*	[22]
pAX-BtPS	pAX01 + *motPS* from *B. trypoxylicola*	This study
pAX-BaPS	pAX01 + *motPS* from *B. alcalophilus*	This study
pAX-tpPS	pAX01+tp*-motPS* (*motP* from *B. trypoxylicola*, *motS* from *B. pseudofirmus*)	This study
pAX-taPS	pAX01+ta*-motPS* (*motP* from *B. trypoxylicola*, *motS* from *B. alcalophilus*)	This study
pAX-ptPS	pAX01+pt*-motPS* (*motP* from *B. pseudofirmus*, *motS* from *B. trypoxylicola*)	This study
pAX-paPS	pAX01+pa*-motPS* (*motP* from *B. pseudofirmus*, *motS* from *B. alcalophilus*)	This study
pAX-atPS	pAX01+at*-motPS* (*motP* from *B. alcalophilus*, *motS* from *B. trypoxylicola*)	This study
pAX-apPS	pAX01+ta*-motPS* (*motP* from *B. alcalophilus*, *motS* from *B. pseudofirmus*)	This study

## Supplementary Materials

The following are available online at https://www.mdpi.com/2218-273X/10/5/691/s1, Table S1: Primer list for this study.
