# Supplementary material for: MotP Subunit is Critical for Ion Selectivity and Evolution of a K+-Coupled Flagellar Motor
Source: Biomolecules. 2020 Apr 29;10(5):691. doi: 10.3390/biom10050691 (PMC7277484; doi:10.3390/biom10050691)
Supplement: Supplementary file 1 [file biomolecules-10-00691-s001.pdf]

Table S1. Primers used in this study.

Primer	Sequence（5′ → 3′）	Accession number and corresponding sequence
T7-F	tgtaatacgactcactatcgg	U25268.1 (2249-2269)
SP6-R	atttaggtgacactatagaat	U25268.1 (153-173)
Btr-1	acataataaagcgcgctcgg	LC532380 (745-764)
Ba-ccpA-1-F	gttaaccttgcggacaagcc	JN56194 (1113-1141)
Ba-pfs-2-R	gctgcatttacttttcctactcc	JN561694 (2395-3317)
Bt-motP-EcoRI-F	aatagaAttCggagattaattcaatcaggg	LC532380 (279-308)
Bt-motS-XbaI-R	cctattttcatttctaGAacctcacagcatcc	LC532380 (2010-2041)
Bt-motP-SacII-F	tagaCCGCggagattaattcaatcaggg	LC532380 (281-308)
Bt-motS-SacII-R	cctaCCGCGGtttctaacacctcacagcatcc	LC532380 (2010-2041)
Bp-MotP-SacII-F	cctaaagttccgCgGaccaaaaccg	AL009126.3 (1435315-1435291)
Bp-MotS-SacII-R	cattcatCCgCGGattactgc	AL009126.3 (2138860-2138881)
Ba-MotP-BamHI-F	gtcgtGGATccacaccgtatagaatttag	JN561694.1 (1378-1377)
Ba-MotS-SacII-R	ggaatattgtctccGcgggtcttaataag	JN561694.1 (3015-2987)
BtP-BpS-(F)	ggtgatcaagatgttgaagcgtagatgaagcgtaggcgacgccagcaagag	LC532380 (1097-1120), U91841.2 (1711-1734)
BtP-BpS-(R)	ctcttgctggcgtcgcctacgcttcatctacgcttcaacatcttgatcacc	LC532380 (1097-1120), U91841.2 (1711-1734)
BpP-BtS-(F)	gggatgaagacaatgaagcgtagatgttgaagcgtaggcgtcaacaagttgataaagg	LC532380 (1105-1141), U91841.2 (1699-1721)
BpP-BtS-(R)	cctttatcaacttgttgacgcctacgcttcaacatctacgcttcattgtcttcatccc	LC532380 (1105-1141), U91841.2 (1699-1721)
BpP-BaS-(F)	gggatgaagacaatgaagcgtagatgctgaggcgtagaaaacaagtacctg	U91841.2 (1699-1721), JN561694.1 (2257-2284)
BpP-BaS-(R)	caggtacttgttttctacgcctcagcatctacgcttcattgtcttcatccc	U91841.2 (1699-1721), JN561694.1(2257-2284)
BaP-BpS-(F)	gggtgaacaaaatgctgaggcgtagatgaagcgtaggcgacgccagcaagag	JN561694.1(2247-2271), U91841.2 (1711-1734)
BaP-BpS-(R)	ctcttgctggcgtcgcctacgcttcatctacgcctcagcattttgttcacc	JN561694.1 (2247-2271), U91841.2 (1711-1734)
BtP-BaS-(F)	ggtgatcaagatgttgaagcgtagatgctgaggcgtagaaaacaagtacctg	LC532380 (1097-1120), JN561694.1 (2257-2284)
BtP-BaS-(R)	caggtacttgttttctacgcctcagcatctacgcttcaacatcttgatcacc	LC532380 (1097-1120), JN561694.1 (2257-2284)
BaP-BtS-(F)	ggtgaacaaaatgctgaggcgtagatgttgaagcgtaggcgtcaacaagttgataaagg	JN561694.1 (2247-2271), LC532380 (1105-1141)
BaP-BtS-(R)	cctttatcaacttgttgacgcctacgcttcaacatctacgcctcagcattttgttcacc	JN561694.1 (2247-2271), LC532380 (1105-1141)
pAX01-F	ccgctcatgagacaataaccc	AB5177281.1 (7398-7348)
pAX01-R	gggggaaatgacaaatggtcc	D50399.1 (3236-3216)

Amino acid substitution mutation sites are shown in capital letters in the base sequence of each primer, and restriction enzyme recognition sites are shown in red. Minus strand primers are underlined in the corresponding sequence.
